# The Effects of Resistance Exercise Training on Strength and Functional Tasks in Adults With Limb-Girdle, Becker, and Facioscapulohumeral Dystrophies

**DOI:** 10.3389/fneur.2019.01216

**Published:** 2019-11-19

**Authors:** Emma L. Bostock, Dawn N. O'Dowd, Carl J. Payton, Dave Smith, Paul Orme, Bryn T. Edwards, Christopher I. Morse

**Affiliations:** ^1^Musculoskeletal Physiology Research Group, School of Science and Technology, Nottingham Trent University, Nottingham, United Kingdom; ^2^Research Centre for Musculoskeletal Science & Sports Medicine, Department of Sport and Exercise Sciences, Manchester Metropolitan University, Manchester, United Kingdom; ^3^The Neuromuscular Centre, Winsford, United Kingdom

**Keywords:** functional tasks, Becker, Limb-Girdle, Fascioscapulohemeral, muscular dystrophy, resistance training

## Abstract

**Background:** The inclusion of resistance training in the treatment and management of muscular dystrophy has previously been discouraged, based on mainly anecdotal evidence. There remains a lack of experimental investigation into resistance training in individuals with muscular dystrophy. The aim of the current study was therefore, to determine the effect of a 12-week resistance training programme on muscle strength and functional tasks in ambulatory adults with muscular dystrophy.

**Methods:** Seventeen ambulatory adults with muscular dystrophy (Facioscapulohumeral muscular dystrophy: *n* = 6, Limb-Girdle muscular dystrophy: *n* = 6, Becker muscular dystrophy: *n* = 5) were recruited for this study. Participants attended three testing sessions: one session at baseline, one session after a 12-week control period and one session after a 12-week resistance training period. Each testing session consisted of measurements of isometric knee extensor and knee flexor maximum voluntary contraction (MVC) torque (Cybex dynamometer). Participants also completed a timed sit-to-stand, a four steps-stair ascent, and a four steps-stair decent. The 12-week resistance training period consisted of two supervised sessions a week. Each training session included a 5-min warm-up, a step-up exercise, free-standing or assisted squats, knee flexion and knee extension exercises, and an additional 6 single-joint exercises specific to each individual's needs.

**Results:** Knee flexor MVC torque increased by 13% after the 12-week resistance training programme (*p* < 0.05), with no change over the control period. Knee extensor MVC torque did not significantly change after the training programme or the control period. Time taken to complete sit-to-stand, stair ascent and stair descent all decreased (improved) following the 12-week training programme (*p* < 0.05).

**Conclusions:** A twice-a-week, 12-week, resistance training programme resulted in increased knee flexion strength and improvements in functional tasks in ambulatory adults with muscular dystrophy. This provides support for the inclusion of resistance training in the treatment programmes for these forms of muscular dystrophy.

## Introduction

Muscular dystrophy (MD) is an umbrella term for a group of inherited myopathic conditions, caused by mutations in a number of genes (1). This incorrect or missing genetic information alters the structure of the proteins within the sarco-glycan complex (among others), leading to a progressive decline in muscle strength in the affected muscles (2). The combined prevalence of all muscular dystrophies ranges between 19.8 and 25.1 per 100,000 individuals in the UK (3). Despite genetic differences, and condition-specific presentation of physical impairment (1), all MDs result in progressive muscle weakness and a reduced ability to complete functional daily tasks, e.g., sit-to-stand, stair climbing and walking (4). MD encompasses a range of disorders, including Becker (BMD), Limb-Girdle (LGMD), and Facioscapulohumeral (FSHD), which unlike the 100% of adults with Duchenne (DMD) who are non-ambulatory, approximately 50% of adults with these conditions are able to complete walking tasks (5).

Developing or maintaining muscular strength is important for physical health and functional ability in healthy adults (6). Of particular interest to the completion of functional tasks is the development of strength in the muscles of the lower limb; it is known for example that knee extension and knee flexion maximum voluntary contraction (MVC) torque contribute to stair climbing in other clinical conditions (7–9). Within myotonic dystrophy and boys with DMD the strength of lower limb muscles has been associated with timed motor tasks (10), fall frequency (11), and 9 m run time (12). In terms of adaptations with training, it is well-established that resistance training increases lower limb muscle strength and physical function in a healthy population (13, 14). These benefits of resistance training are also known to be effective in populations characterized with muscle weakness, such as elderly individuals (15), stroke patients (16), and adults with multiple sclerosis (17). It would therefore seem logical that lower limb resistance training may benefit individuals with MD by maintaining or improving muscle strength and functional ability.

Despite the known benefits of resistance training in other populations, strength training has long been discouraged in MD due to historical fears that it may have detrimental effects (18). For many years it has been believed that a weak muscle is more susceptible to overwork damage, because it is already functioning close to its maximum capacity (19, 20). This dated perspective has received extremely limited scientific scrutiny and the value of resistance training in MD remains controversial, despite the model for a harmful effect being based on a murine form of MD and anecdotal evidence (21). A systematic review and meta-analysis of resistance training in MD retrieved only five studies (18). Those studies included children with DMD (22), adults with Myotonic dystrophy (23–25), and adults with FSHD (26). A sixth, more recent training study has been published on adults with LGMD and BMD (27). Most studies that have measured the effect of resistance training on isometric strength in those with MD have found no significant benefit following training in the knee extensors (22–24), knee flexors (24), elbow flexors, and dorsi flexors (26). In contrast, Sveen et al. (27) reported 60 and 35% increases in elbow and knee extensor muscle strength respectively, following 24 weeks of resistance training in adults with LGMD and BMD. The meta-analysis by Gianola et al. (18) also revealed that resistance training had no significant effect on multiple measures of physical function, such as time to stand up from a seated position, time to ascend stairs and time to descend stairs. The non-significant outcomes summarized by Gianola et al. (18) are likely due to small numbers of participants [e.g., *n* = 6 (23)], and the fact that a positive training adaptation may be unlikely in a condition defined by continuous declines in strength. Given the progressive nature of MD, the ability to maintain strength with resistance training could be meaningful, despite not reaching statistical significance.

There currently remains no answer to whether resistance training is beneficial, null or detrimental in adults with MD. Numerous limitations in previous research highlight the need for further studies that focus on the effect of resistance training on the strength of various muscles, the inclusion of functional measures and the use of appropriate control groups. Therefore, the aim of the present investigation was to determine the effect of a twice weekly resistance training programme of 12 weeks' duration on muscle strength and functional tasks in ambulatory adults with MD.

## Materials and Methods

### Participants

Seventeen adults previously diagnosed with MD (FSHD, LGMD, and BMD) were recruited from The Neuromuscular Centre (Winsford, UK). All participants were considered ambulatory (as described below). The participant characteristics of each MD sub-group and a grouped average for MD are described in Table 1. All participants provided written informed consent before taking part in the study, which was approved by the local Ethics Committee of Manchester Metropolitan University. All procedures complied with the World Medical Association Declaration of Helsinki (28).

**Table 1:** Participant characteristics and anthropometric measurements at baseline (PRE) for muscular dystrophy (MD) grouped and separated into muscular dystrophy sub-groups.

	**Muscular dystrophy grouped**	**FSHD**	**LGMD**	**BMD**
*N*	17	6	6	5
Sex	Male: 13, Female: 4	M: 4, F: 2	M: 4, F: 2	M: 5
Age (years)	44 ± 11	43 ± 12	47 ± 11	40 ± 8
Stature (m)	1.77 ± 0.09	1.80 ± 0.05	1.71 ± 0.09	1.80 ± 0.09
Body mass (kg)	87.9 ± 17.3	90.1 ± 14.9	82.0 ± 19.4	92.3 ± 19.0

*Data are presented as mean ± SD*.

All participants had not previously undertaken structured resistance training, were functionally active (ambulatory) and self-reported that they did not undertake more than 1 h of intense physical activity or 3 h of low-moderate physical activity per week. The participants were all receiving regular (weekly, bi-weekly, or monthly) physiotherapy treatment at The Neuromuscular Centre, that involves passive mobility activities lasting ~1 h. All participants were able to walk at least seven meters with or without assistive walking devices, were in otherwise good health and without any uncontrolled co-morbidity or cardiac issues. Consistent with previous studies that have “grouped” multiple MDs for analysis [e.g., BMD and LGMD (27), FSHD, and LGMD (29)], we grouped all the participants as “adults with muscular dystrophy.” This is discussed later within the limitations section of the discussion, and represents the rationale for the within group study design (see section Materials and Methods below).

### Procedures

**Figure 1:**
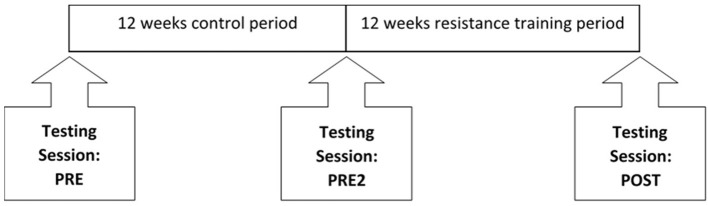
Schematic of study design.

All participants were screened for eligibility during a face-to-face meeting or via e-mail, before attending three testing sessions at the university. Baseline measurements were taken at PRE, followed by a second testing session 12-weeks after a control period (PRE2) and a third testing session within 2 weeks of completing a 12-week resistance training programme (POST, Figure 1). Due to the progressive nature of and inherent variance within these conditions, participants acted as their own controls. The single-arm non-crossover design where the participant acts as his or her own control, although not as robust as randomized control exercise trials, is advocated for longitudinal interventions in populations such as the present one (30). This training study design is also consistent with previous resistance training studies (31), and seeks to overcome the possible variability of the training response within a population. During the 12-week control period, participants were asked to maintain their habitual physical activity levels. All testing sessions were identical between participants and between testing sessions, with the participant having refrained from consuming caffeine and alcohol in the 12 h prior to the start of testing. Participants also completed three timed functional tests at The Neuromuscular Centre, within 1 week of each testing session.

### Resistance Training Programme

**Figure 2:**
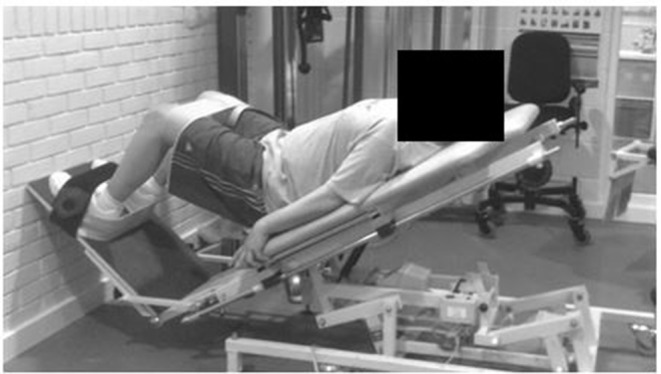
Adapted physiotherapy treatment bed to produce a squat like movement.

Each participant attended two supervised resistance training sessions each week over the 12-week training period (24 training sessions in total). Participants received a personalized training plan that was progressive and designed specifically to their individual needs and capabilities. All training programmes included two warm-up exercises and four consistent resistance exercises, in addition to six individualized exercises. All training sessions started with a 5-min warm up on a seated cross-trainer, cycle ergometer or a rowing machine, followed by 5–10 min of balance training on a Wii Fit video game balance board (Nintendo of America Inc., Redmond, WA). Following the warm-up, all participants completed two multi-joint exercises: a step-up exercise onto a box of various and progressive heights and freestanding or assisted squats. Assisted squats were either (i) a squat exercise with a Swiss ball placed between the participant's lower back and the wall to aid balance in the posterior direction; (ii) an exercise on an adapted physiotherapy treatment bed that allowed participants to complete a movement similar to that of a squat (simultaneous flexion and extension of the ankle, knee, and hip joint) with a reduced proportion of body weight involved in the movement (Figure 2). Whilst the participants lay on the bed, the gradient of the plinth could be increased from a horizontal position to a full, but supported, standing position, or any gradient in between. The angle of the plinth, and therefore the proportion of body weight involved in the movement, was reviewed and increased (if appropriate) every 3 weeks. The final two exercises included within all programmes were knee flexion and knee extension on a seated leg curl and extension machine (Pro Heavy Duty, XS Sports). Participants were seated on the leg curl and extension machine and then placed their legs either behind or in front of the padded bar (depending on whether they were working in the direction of flexion or extension). Participants were asked to either flex or extend their legs as far as possible with resistance of the bar and additional weights attached to this bar. Participants then completed six lower-body additional single-joint resistance exercises, based on a needs analysis completed at PRE. These additional exercises included hip flexion, hip extension, plantar flexion, dorsi flexion, hip abduction, hip adduction, hip internal rotation or hip external rotation. For all exercise's participants were instructed to complete each movement through their maximum range.

Initially, two sets of 10 repetition maximums (RM) per exercise were performed, with the number of sets increasing to three after 3 weeks. One-minute's rest was given between exercise sets. Participants were instructed to complete exercises at a moderate repetition velocity (2 s concentric: 4 s eccentric), a protocol previously shown to induce greater 1RM strength gains (39 vs. 15%) than slower repetition velocity protocols (32). The training weight of 10RM was reviewed and adjusted (if the current load could be lifted for two repetitions over the desired amount) every 3 weeks during the training period. This training volume and load are in line with those recommended by the American College of Sport Medicine (6) and to those used in other MD training studies (23, 24, 27). In circumstances where participants could not complete an exercise through its full range of motion, due to the weight of their body or limb, the investigator provided assistance manually or with rubber exercise bands, or the movement was completed in a gravity neutral/assisted position (e.g., side lying knee flexion).

### Anthropometric Measures

Stature and body mass were measured using a wall-mounted stadiometer (Harpenden, Holtain Crymych, UK) and digital scales (Seca model 873, Seca, Germany), respectively.

### Knee Flexor and Extensor Torque

Isometric MVC torque was measured using an isokinetic dynamometer (Cybex Norm, Cybex International Inc., NY, USA) for knee flexion and knee extension. Participants sat on the dynamometer chair with a hip angle of 85° and were secured with straps around the shoulders, hips, and thigh. The cuff of the lever arm was attached proximal to the medial malleolus on the dominant leg. The dominant limb was determined via self-report, in answer to the following question: which leg would you use to stop a football that was rolling toward you? Participants completed five submaximal warm-up contractions of knee flexion, followed by five submaximal warm-up contractions of knee extension. Subsequently, at an angle of 70° knee flexion, participants completed two maximal isometric contractions of knee flexion, followed by two maximal isometric contractions of knee extension, on the dominant leg. Participants were instructed to contract maximally for approximately 4 s, until verbally cued to relax. Verbal encouragement was given during MVCs and a 3-min rest period was given between each MVC. MVC torque was presented in real-time to participants and recorded on an Apple computer (Cupertino, CA, USA) via an A/D converter (Biopac Systems, Santa Barbara, CA). Torque measurements were processed offline using Acknowledge software (Version 3.9.2). Baseline torque was removed from the trace and then peak torque was measured. The highest torque produced during knee extension and knee flexion was taken as MVC peak torque.

### Functional Tasks

Each participant completed three timed functional tests at The Neuromuscular Centre. These tests included a sit-to-stand, a stair ascent and a stair descent. The sit-to-stand test required participants to rise from a seated position to standing from a standardized chair. The stair ascent required participants to climb four steps and the stair descent required the participants to climb down the same set of four steps. Participants were asked to complete all tasks as fast and safely as possible. Participants were asked not to use the arms of the standardized chair, unless necessary. Participants were permitted to use the arms of the chair or the handrails of the stairs if necessary, but this was kept consistent throughout all testing sessions. Participants completed the sit-to-stand, stair ascent, and stair descent tests at PRE, PRE2, and POST.

### Statistics

IBM SPSS Statistics 24 software was used to analyse the data. All data are presented as mean ± SD. Statistically significant differences were accepted at α ≤ 0.05. Body mass, knee flexion torque and knee extension torque violated the parametric assumption of normal distribution (Shapiro–Wilk test, p < 0.05), all other variables were normally distributed. Differences between the repeat testing sessions (PRE, PRE2, and POST) were analyzed using a one-way repeated measures ANOVA with Bonferroni corrected *post-hoc* pairwise comparisons. If the data did not pass Mauchly's test of sphericity (*p* < 0.05), a Greenhouse-Geisser correction was applied. Variables that violated normal distribution were compared between repeat testing sessions using the Friedman test, with *post-hoc* Wilcoxon signed-rank pairwise comparisons, where appropriate.

For within participant comparisons, data are grouped as one MD group rather than three individual MD sub-groups, this is consistent with other training studies in MD (27). Further, there were no differences in primary outcome measures between MD sub-groups (*p* > 0.05).

For between MD sub-group comparisons at PRE, data were analyzed using a one-way between group ANOVA with Bonferroni corrected *post-hoc* pairwise comparisons for variables that met the assumption of normal distribution. Variables that violated normal distribution were compared between groups at PRE, using the Kruskal–Wallis test, with *post-hoc* Mann–Whitney *U* pairwise comparisons.

## Results

### Demographic and Anthropometric Measures

Age, stature and body mass were not significantly different between MD sub-groups at PRE (*p* > 0.05; Table 1). Body mass and stature were not significantly different between all three repeat testing sessions (PRE: 87.9 ± 17.3 kg; PRE2: 89.2 ± 19.0 kg; POST: 88.1 ± 18.1 kg and PRE: 176.8 ± 8.6 cm; PRE2: 176.9 ± 8.6 cm; POST: 177.0 ± 8.4 cm, respectively).

Thirteen of the seventeen participants completed all 24 training sessions, two participants completed 23 sessions, one participant completed 22 sessions and one participant completed 18 training sessions of the 24. Zero adverse events were reported to investigators by the 17 participants, but one participant fell during a training session. However, the participant sustained no lasting injuries and continued with the resistance training programme the following week. Two additional participants started the training programme but withdrew, one dropped out after the second exercise session and the second dropped out after the fifth exercise session (data from these participants were omitted from all results). The reasons stated for dropping out were (1) a lack of time; (2) flare up of an injury that was sustained before the resistance training programme.

### Knee Flexor and Knee Extensor Torque

Knee flexor and knee extensor MVC torques were not significantly different between MD sub-groups at PRE (*p* > 0.05, Table 2). Knee flexor MVC torque was significantly different between testing points (*p* = 0.005). Knee flexor MVC torque was not different between PRE and PRE2 (*p* > 0.05). Knee flexor MVC torque increased by 13% from PRE2 to POST (*p* = 0.004). Knee extensor MVC torque was not significantly different between testing points (*p* = 0.085).

**Table 2:** The effect of resistance training on knee flexor and knee extensor torque in muscular dystrophy.

	**PRE**	**PRE2**	**POST**
Knee Flexor MVC Torque (N.m)	35.6 ± 31.3	36.5 ± 34.7	41.4 ± 37.7^*^^#^
Knee Extensor MVC Torque (N.m)	81.0 ± 84.0	82.7 ± 89.1	88.0 ± 94.7

*Data are presented as mean ± SD. MVC, maximal voluntary contraction*.

* *Denotes a significant difference from PRE*.

# *Denotes a significant difference from PRE2*.

### Functional Tasks

The time taken to complete the stair descent was not significantly different between MD sub-groups at PRE. The time taken to complete the sit-to-stand and stair ascent tests was significantly longer in the BMD group compared to the FSHD group at PRE (*p* = 0.046; and *p* = 0.011, respectively), with no differences between the other groups.

Sit-to-stand time was significantly different between testing sessions (*p* < 0.001, Table 3). Specifically, sit-to-stand time decreased by 36% from PRE2 to POST (*p* = 0.002). Sit-to-stand time was not different between PRE and PRE2. Stair ascent time was significantly different between testing sessions (*p* = 0.002, Table 3). Stair ascent time decreased by 18% from PRE2 to POST (*p* = 0.017). Stair ascent time did not change significantly between PRE and PRE2. Stair descent time was significantly different between testing sessions (*p* < 0.001; Table 3). Stair descent time decreased by 24% from PRE2 to POST (*p* = 0.003). Stair descent time did not change significantly between PRE to PRE2.

**Table 3:** The effect of resistance training on functional tasks in muscular dystrophy.

	**PRE**	**PRE2**	**POST**
Sit-to-stand (s)	3.64 ± 1.64	3.91 ± 1.95	2.49 ± 1.43^*^^#^
Stair ascent (s)	7.38 ± 3.30	7.18 ± 3.44	5.89 ± 2.48^*^^#^
Stair descent (s)	5.09 ± 1.99	5.61 ± 2.31^*^	4.28 ± 1.50^*^^#^

*Data are presented as mean ± SD*.

* *Denotes a significant difference from PRE*.

# *Denotes a significant difference from PRE2*.

## Discussion

The main findings from the present study show increased muscle strength and improvements in functional tasks in response to a twice-a-week, 12-weeks, resistance training programme in ambulatory adults with MD. Our data show a significant improvement in knee flexor MVC torque after resistance training, but no significant change in knee extensor MVC torque. The time taken to complete a sit-to-stand, stair descent and stair ascent all decreased (improved) in response to the 12-weeks of resistance training in adults with MD.

Knee flexor MVC torque increased by 13% from immediately before to post completion of the resistance training programme (PRE2 to POST), with stable values during the 12-week control period. In contrast, Lindeman et al. (24) found no increase in knee flexion MVC torque after 24 weeks of resistance training, which involved three sessions a week of home-based exercises. It is important to note that in the previous study exercise sessions were unsupervised, the programme targeted only four lower limb movements and the participants had Myotonic dystrophy, compared to supervised sessions, numerous lower limb movements and participants with FSHD, LGMD, and BMD in the current study. The present improvements in knee flexion MVC torque following resistance training should relate to functional benefits to those with MD. Previous data from other clinical conditions shows knee flexion MVC to be correlated to stair climb performance (7–9). Future studies are needed to assess the relative importance of muscle weakness, strength gains and functional tasks in adults with MD.

There was no change in knee extensor MVC torque in the present study; although the direction of change was positive it did not reach significance. In contrast to this, Sveen et al. (27) reported a 35% increase in knee extensor torque following 24 weeks of low-intensity resistance training in adults with LGMD and BMD. In the previous study however, muscle strength was measured using 1-repetition maximum strength compared to isometric MVC torque in the current study. The length of intervention of the resistance training may have also contributed to the discrepancies between the current study (12-weeks) and the previous study (6 months) (27). It is also important to highlight the large standard deviation in the knee extensor torque values in the present study, due to the heterogeneity of BMD, LGMD, and FSHD. This could have contributed to the lack of significant difference found in knee extensor MVC torque in the current study, and it reflects the nature of the MD conditions.

In terms of functional ability, the current study resulted in improvements across all the functional tasks. These improvements (reductions) in time taken to complete a sit-to-stand, stair ascent and stair descent ranged between 18 and 36% from immediately before to after the resistance training programme (PRE2 to POST). These improvements have not been reported previously, for example in their systematic review Gianola et al. (18) identified no effect of exercise on any of the functional tasks recorded previously in adults with different forms of MD. In contrast to these previous studies that trained isolated muscle groups and focused on only a few joint movements, we adopted a more functional approach to the exercise training. It is therefore pertinent to suggest that the inclusion of “posterior chain” exercises that resemble the challenges of everyday life should form the basis of resistance training in adults with MD to ensure improvements in functional tasks that are reflected by the outcome measures of sit-to-stand and timed walks.

The limited research in resistance training and MD has not consistently shown improvements in muscle strength [e.g., no change in knee extensor (22–24), knee flexor (24) or dorsi flexor (26) strength] or physical function [e.g., no change in stair ascent or rise from a chair (24)] with resistance training in muscular dystrophy (18). In contrast, the resistance training programme adopted in the present study improved knee flexion MVC torque and physical function in adults with MD. These previous studies only trained isolated muscle groups and movements. In contrast to this, the current study involved several isolated movements of the lower-limb, along with multi-joint movements (e.g., squats). This alone may explain the positive results observed in the current study compared to previous studies (33). In addition to this, previous studies have required participants to exercise at home, unsupervised. The one-to-one supervised nature of the current resistance training intervention may also explain the improvements seen in the current study (34), which were not seen in previous studies, and suggests a need for greater awareness and provision of targeted, supervised exercise for adults with muscular dystrophy as a way of maintaining physical function as their condition develops.

Although reporting improvements in strength and functional ability in the current study, it is important to highlight the limitation of grouping the three types of MD together. This study provides strong evidence for resistance training in adults with MD, but future studies should look to examine the effects of resistance training on different MDs in isolation to allow for tailored exercise prescription. To date there are no data that suggest training adaptations are different between the MD populations included in the present study. Indeed, a discussion on the differences to the acute responses to exercise that may inform MD specific training adaptations is also not possible. However, based on damage response to high intensity endurance exercise, it seems that there may be differences in the recovery from exercise in LGMD, BMD, and FSHD (35). This however, includes numerous leaps of interpretation to be able to inform the possible training differences that may be seen between different MD groups. Ultimately, however, if we consider the variance of the two main outcome measures in the present study, knee extension MVC and knee flexion MVC, the variability within the MD groups at PRE1 is as large as that between the MD groups. It is therefore pertinent to state that future research should build on the positive effect of resistance training in the present study, to consider whether differences to training adaptation may exist between the MD conditions, or indeed whether that is relevant considering that the positive functional adaptations described here are meaningful to those with MD. It is also important to examine the frequency and intensity of resistance training in these populations. This study showed that a resistance training programme similar to the one in this study can be safely undertaken in adults with FSHD, BMD, and LGMD, but an important question remains regarding how much resistance training is too much in these conditions.

The findings of the current study provide support for the inclusion of resistance training in the management and treatment programmes of adults with MD, despite previous medical concerns. Resistance training is a feasible approach to maintain or improve muscle strength and physical independence in adults with muscular dystrophy. In conclusion, we have demonstrated the beneficial effects of a resistance training programme compared to a control period on knee flexor MVC torque and functional tasks in ambulatory adults with FSHD, LGMD, and BMD.

## Data Availability Statement

The raw data supporting the conclusions of this manuscript will be made available by the authors, without undue reservation, to any qualified researcher.

## Ethics Statement

The studies involving human participants were reviewed and approved by The local Ethics Committee of Manchester Metropolitan University. The patients/participants provided their written informed consent to participate in this study.

## Author Contributions

EB and DO'D contributed to study design, collected the data and contributed to the writing of the manuscript. CP, CM, and DS supervised the project and reviewed the manuscript. BE and PO contributed to exercise prescription and participant identification.

### Conflict of Interest

The authors declare that the research was conducted in the absence of any commercial or financial relationships that could be construed as a potential conflict of interest.
